# From bench to bed: the tumor immune microenvironment and current immunotherapeutic strategies for hepatocellular carcinoma

**DOI:** 10.1186/s13046-019-1396-4

**Published:** 2019-09-09

**Authors:** Yaojie Fu, Shanshan Liu, Shan Zeng, Hong Shen

**Affiliations:** 1 Department of Oncology, Xiangya Hospital, Central South University, Changsha, 410008 Hunan China; 2 National Clinical Research Center for Geriatric Disorders, Xiangya Hospital, Central South University, Changsha, 410008 Hunan China; 3 Key Laboratory for Molecular Radiation Oncology of Hunan Province, Xiangya Hospital, Central South University, Changsha, 410008 Hunan China

**Keywords:** Hepatocellular carcinoma (HCC), Immunotherapy, Oncolytic virus, Immune checkpoint blockade (ICB), Adoptive cell transfer

## Abstract

Hepatocellular carcinoma (HCC) ranks the most common primary liver malignancy and the third leading cause of tumor-related mortality worldwide. Unfortunately, despite advances in HCC treatment, less than 40% of HCC patients are eligible for potentially curative therapies. Recently, cancer immunotherapy has emerged as one of the most promising approaches for cancer treatment. It has been proven therapeutically effective in many types of solid tumors, such as non-small cell lung cancer and melanoma. As an inflammation-associated tumor, it’s well-evidenced that the immunosuppressive microenvironment of HCC can promote immune tolerance and evasion by various mechanisms. Triggering more vigorous HCC-specific immune response represents a novel strategy for its management. Pre-clinical and clinical investigations have revealed that various immunotherapies might extend current options for needed HCC treatment. In this review, we provide the recent progress on HCC immunology from both basic and clinical perspectives, and discuss potential advances and challenges of immunotherapy in HCC.

## Background

Hepatocellular carcinoma (HCC) represents the most common type of primary liver cancer, with a global incidence of 500,000 new cases per year [1]. HCC is closely associated with chronic liver inflammation and some well-known risk factors, including chronic HBV and HCV infections, alcohol consumption, diabetes mellitus and several metabolic diseases [2]. The current therapeutic options available for HCC, such as transarterial chemoembolization (TACE), radiofrequency ablation, surgical resection and transplantation, are only curative for some patients in early stages. Other more effective approaches emerged in the past few years, such as tyrosine kinase inhibitors (TKIs) targeting angiogenesis (e.g. Sorafenib, lenvatinib, regorafenib) [3], clinically tested selective Cyclin dependent kinase 5 and 4/6 (Cdk5, Cdk4/6) inhibitors (Dinaciclib & Palbociclib) [4–6], and highly selective fibroblast growth factor receptor 4 (FGFR4) inhibitor H3B-6527 [7, 8], which pre-clinically and clinically show encouraging efficacy and have been rigorously pursued for advanced HCC.

The liver is a ‘tolerogenic’ organ that can arouse its immune responses to prevent undesirable pathogen attack and tumor initiation. However, as a typical inflammation-linked tumorigenesis, immune evasion is one of the features occurring during the initiation and evolution of HCC [9]. A number of immune suppressor mechanisms, including intratumoral accumulation of immunosuppressive cell populations, defective antigen presentation and activation of multiple inhibitory receptor-ligand pathways, favor tolerance over immunity, and promote progression of HCC [10, 11]. The magnitude of immune suppression in the tumor microenvironment (TME) is closely correlated with poor prognosis in HCC patients. Hence, for better arousing anti-tumor immunity, more details about suppressed immune landscape of HCC urgently needs to be elucidated.

### The intricate immune network in TME of HCC

**Fig. 1:**
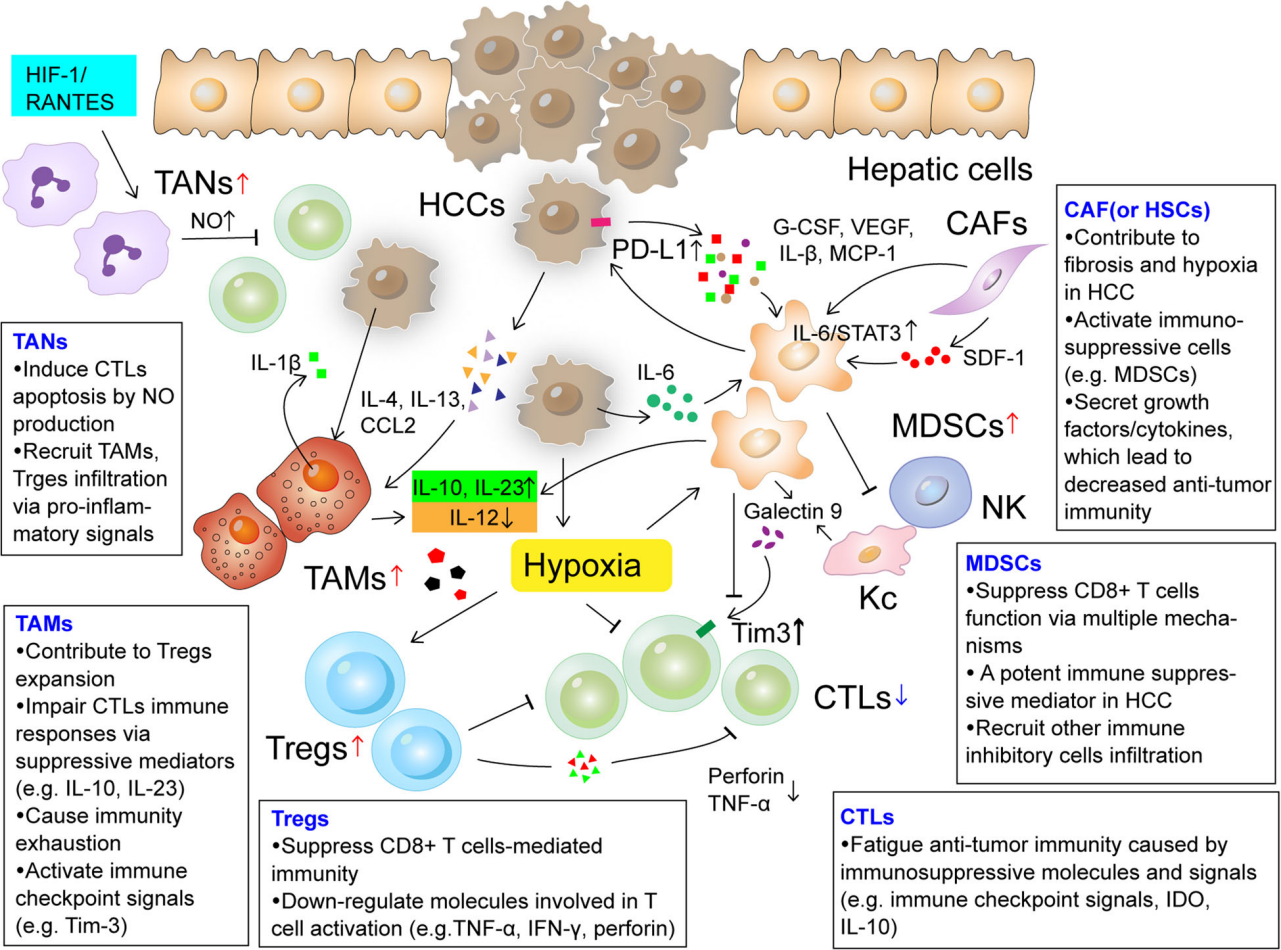
The landscape of immunosuppressive tumor microenvironment of HCC. Diverse suppressive immune cell subsets infiltration, regulatory secretions and some inhibitory signaling mediate HCC immune evasion. (Notes: Tregs: regulatory T cells; TAMs: tumor-associated macrophages; TANs: tumor associated neutrophils; CTLs:cytotoxic T lymphocytes; CAF: cancer associated fibroblast; MDSCs: myeloid- derived suppressor cells; HSCs: hepatic stellate cells; NK: natural killer cell; KC: Kupffer cell)

The HCC tumor microenvironment (TME) is a dynamic system, which comprises cancer cells, the intricate cytokine environment, extracellular matrix, immune cell subsets and other components [12]. It’s well established that the immune landscape of HCC has a strong suppressor feature. In this complex network, the pro-tumorigenic immune response, mediated by diverse immunosuppressive cell subsets, secretions and signaling, plays a pivotal role in driving immune evasion [13] (Fig. 1**.**). Moreover, ‘fatigue’ of anti-tumor immunity also contributes to tumor tolerance and progression. Here, we discuss new advances in the immunosuppressive picture of HCC.

### Representative immunosuppressive components in TME of HCC

#### Myeloid-derived suppressor cells (MDSCs)

MDSCs is a heterogeneous population of immature myeloid cells (IMCs), which are expanded in pathological conditions and up-regulate expression of immune suppressive factors, such as arginase and inducible nitric oxide synthase (iNOS or NOS2) [14]. Various tumor originated cytokines, such as G-CSF, GM-CSF, VEGF, MCP-1 and IL-1β, have been demonstrated to induce MDSCs infiltration [15]. Cell cycle related kinase (CCRK) represents a novel signaling target for cancer immunotherapy [16]. Emerging evidence also indicates the hepatoma-intrinsic CCRK upregulates interlukin-6 (IL-6) production through EZH2/NF-κB signaling, which consequently induce MDSCs accumulation in TME [17]. Hepatic carcinoma related tumor-associated fibroblasts (TAFs), a stromal part in HCC, can induce peripheral blood monocyte migration and differentiation into CD14 ^+^ HLA-DR ^−/low^ MDSCs by SDF-1α. TAFs mediate the generation of MDSCs via IL-6/STAT3 signaling [18]. In addition, local hypoxia has been identified as a key regulator that can promote MDSCs accumulation through the Chemokine C-C motif Ligand 26 (CCL26)/CX_3_CR1 pathway [19]. Hypoxia-inducible factor 1α (HIF-1α) mediated ENTPD2 over-expression in HCC cells, has been proven to increase the extracellular level of 5′-AMP, which subsequently recruit MDSCs into TME [20].

As a powerful inhibitory immune modulator, infiltrated MDSCs in HCC damage effector T cells, expand immune checkpoink signaling, decrease NK cell cytotoxicity and cytokine production by diverse mechanisms [21, 22]. The MDSCs in fibrotic HCC tissue are notably correlated with reduced tumor infiltrating lymphocytes (TILs) and elevated tumorigenicity, aggressive phenotype, moreover, whose activation and infiltration contribute greatly to worse survival rate both in mouse model and HCC patients [23]. CD14^+^ HLA-DR ^−/low^ MDSCs can blunt HCC immunity through inducing activation of CD4^+^ CD25^+^ Foxp3^+^ regulatory T cells, inhibiting proliferation and IFN-γ secretion of CD3/CD28-stimulated autologous peripheral blood mononuclear cells (PBMCs) [24]. T cell function is impaired due to competition for energy resources (e.g. arginine and cysteine) with MDSCs [25], as well as involvement of several inhibitory receptor-ligand pathways in MDSCs-mediated immune evasion. Tumor-derived TGF-β triggers recruitment of MDSCs in a CXCL1/2/5- and CXCR2-dependent manner. The infiltrated MDSCs selectively suppress IFN-γ production deriving from NKT cells [26]. MDSCs also can express galectin-9 that binds to TIM-3 on T cells, inducing T-cell apoptosis [27]. In addition, it’s suggested that MDSCs in advanced HCC patients may interact with Kuppfer cells to induce PD-L1 expression, and mediate inhibited cytotoxicity and cytokine release of NK cells through the NKp30 receptor [28]. Taken together, MDSCs exert versatile immunosuppressive effects in HCC. Combined treatment with anti-PD-1/PD-L1 and concomitant targeting MDSCs (such as CCRK inhibition or p38 MAPK inhibitor) may synergistically enhance efficacy to eradicate HCC [17, 23]. In addition, recent evidence suggests radiation and IL-12 combination therapy (RT/IL-12) may elevate anti-tumor immunity in HCC by reducing MDSCs accumulation and the production of reactive oxygen species (ROS) [29]. Hence, MDSCs may serve as a potential target for resetting immunotorelant state in HCC tumors.

#### Tumor-associated macrophages (TAMs)

Macrophages arise from bone marrow-derived circulating monocytes, and then reside in normal tissues. The two polarizing phenotypes M1 and M2, are highly plastic in response to complex stimuli. Substantial clinical data and experimental research confirmed that alternatively activated status macrophages, the M2 phenotype, stimulate tumor initiation, progression and malignant metastasis by various mechanisms [30, 31]. In HCC, some specific populations of the immunosuppressive tumor-associated macrophages (TAMs) have emerged as a research hotspot recently. The well-identified HCC-derived cytokines, such as IL-4, IL-13, CSF-1, CCL2, CXCL12, connective tissue growth factor (CTGF) [32–34], induce TAMs differentiation from CCR2^+^ inflammatory monocytes, alternatively activated macrophages (AAMs) infiltration, then reduce innate or adaptive immunity [34, 35]. Osteopontin (OPN) expressed by HCC cells not only has a positive association with PD-L1 expression in HCC, moreover, it facilitates alternative activation and chemotactic migration of TAMs via CSF1-CSF1R pathway in TAMs [36]. HCC cells originated HIF-1α has been recently suggested to enhance IL-1β release by TAMs via TLR4/TRIF/NF-κB signaling pathway, which fosters EMT and immune evasion of HCC [37]. Crosstalk between MDSCs and TAMs results in decreased production of IL-6, IL-12, down-expression of MHCII, and elevated production of IL-10, a strong inhibitory mediator that impairs downstream CD8+ T cell and NK cell cytotoxicity [38]. TAMs-derived IL-10 also increases intratumoral Foxp3^+^ Tregs frequency, which then suppresses CD4^+^CD25^−^ T cells activation [38, 39]. TAMs in the peritumoral stroma of HCC have been shown to secrete multiple key proinflammatory cytokines (e.g. IL-1β, IL-6, IL-23, and TNF-α) and contribute to the expansion of interleukin-17-producing CD4^+^ T helper 17 cells (Th17), which suppress anti-tumor immunity by over-expressing several activation markers, such as PD-1, CTLA-4, and GITR [40]. In addition, TAMs are highly involved in other immune inhibitory regulations [41, 42]. TGF-β in the TME of HCC promotes the Tim-3 expression on TAMs, subsequently enabling the activated TAMs to facilitate tumor growth and immune tolerance via NF-κB signaling and downstream IL-6 production [43]. TAMs derived IL-6/STAT3 signaling also has been validated to sustain HCC carcinogenesis by promoting its carcinoma stem cells (CSCs)-liked characteristics [44].

Notably, recent evidence indicates that PD-1^−^ TAMs can capture anti-PD-1 monoclonal antibodies (aPD-1 mAbs) through Fcγ receptors (FcγRs) expressed on the surface binding to drug’s Fc domain glycan [45]. This novel investigation indicates that blockade of FcγRs before aPD-1 mAbs administration may substantially improve checkpoint blockade therapy.

#### Tumor associated neutrophils (TANs)

Heterogeneity of TANs is a fundamental property that allows TANs to perform corresponding functions for adaptations to changing microenvironment. Similar to macrophages, neutrophils differently affect tumor biological behaviors depending on their polarization, either anti-tumoral (N1) and pro-tumoral (N2) phenotypes [46]. In some solid tumor models, such as lung cancer, metastatic renal cell cancer (mRCC) and melanoma, it was previously reported that TANs infiltration or neutrophil-lymphocyte ratio (NLR) closely correlate with tumor progression, which can serve as a significant predictor for monitoring patients with advanced tumor receiving anti-PD-1/PD-L1 immunotherapy [47, 48]. TANs activation is modulated by cytokines, such as Type I interferons (IFNs) and TGF-β [49]. TANs mainly suppress anti-tumor immunity via interacting with CD8^+^ T cells, inducing CD8^+^ T cells apoptosis through nitric oxide (NO) production mediated by tumor necrosis factor-α (TNF-α) [50].

The facilitator role of TANs in pathological progression of HCC has become a topic of growing interest in recent years. Clinically, TANs play a key role in driving progression and poor prognosis of HCC, and NLR is an independent predictor of survival after hepatectomy in patients with HCC [51, 52]. The newest discovery shows that loss of hypoxia associated factor, HAF (encoded by *SART1*) results in inappropriate HIF-1 activation, and overproduction of downstream HIF-1 dependent chemokine, RANTES. HIF-1/RANTES upregulation accumulates TANs infiltration, which is associated with non-alcoholic steatohepatitis (NASH) driven HCC initiation and progression [53]. Moreover, recent studies suggested that TANs mediate the intratumoral infiltration of TAMs and regulatory T cells by overproducing some chemokines, such as CCL2 and CCL17, which then contributes to HCC progression, metastasis and resistance to sorafenib treatment [54]. A newly identified positive feedback loop implies that TANs induce HCC stem cell like characteristics via upregulating expression of miR-301b-3p in cancer cells, and maintain hyper-action in NF-kB signaling, lead to higher secretion level of C-X-C motif chemokine5 (CXCL5) and in turn recruit more TANs infiltration [55]. In general, TANs are strongly connected with immunosuppression in HCC, but direct interactions between TANs and other components in HCC tissue and the exact underlying mechanisms behind this regulation in HCC are not yet clear.

### Tumor-infiltrating lymphocytes (TILs)

A high density of tumor-infiltrating lymphocytes (TILs) was once thought to be the host’s immune reaction against cancer. Some early clinical data suggested postoperative HCC patients with high level lymphocytes infiltration, especially T cells, had reduced recurrence and better survival [56]. However, accumulating evidence suggests that the overall degree of TILs in HCC is not capable of mounting effective anti-tumor immunity to control tumor progression [57]. Intrahepatic immune response involves diverse lymphocyte populations, which contribute differently to HCC immune surveillance. The intratumoral balance of regulatory and cytotoxic T cells plays a key role in evaluating the immune state and progression of HCC [57, 58].

#### Regulatory T cell (Treg)

Regulatory T cells (Tregs) can be derived from peripheral blood T lymphocytes, resident T cells and other cellular sources. Its recruitment has been found to be induced by the CCR6 (CC chemokine receptor type 6)–CCL20 (CC motif chemokine 20) axis. Tregs activation is induced by T cell receptor (TCR) engagement concurrent with IL-10 and TGF-β signaling [59]. Apart from activation via pro-inflammatory signals, recent investigations elucidate that long noncoding RNAs (LncRNAs) may play pivotal roles in driving Tregs differentiation and implications during HCC progression [60]. Overexpressed Lnc-epidermal growth factor receptor (Lnc-EGFR) in Tregs binds to EGFR and prevents its ubiquitination by c-CBL, augmenting activation of its downstream AP-1/NF-AT1 axis in Tregs thus to promote immunosuppression in HCC [60]. Moreover, Amphiregulin (AREG), a multifunctional player, may enhance Tregs suppressive function via the EGFR motivation as well [61].

The frequencies of Tregs are associated with HCC invasiveness and have a crucial role in hampering the development of effective anti-tumor responses in HCC [57, 62]. Recent evidence indicates that CD4^+^CD25^+^ Tregs in HCC patients can trigger a compromised immune response through various mechanisms [63]. A typical subset, CD4^+^ CD25^+^ Foxp3^+^ Tregs, may impair CD8^+^ T cells killing capacity via inhibiting the release and production of granzyme A, B (GrA, B), and perforin [64], concurrently, they also selectively suppress certain molecules (such as TNF-α, IFN-γ) involved in CD8^+^ T cell activation [64, 65]. Additionally, high expression of IL-35 in HCC tissue has been implicated positively to correlate with another newly identified subtype, CD39^+^ Foxp3^+^ Tregs infiltration [66], which serves as a better independent predictive indicator for recurrence in HCC patients after curative resection.

#### CD8^+^ cytotoxic T lymphocytes (CTLs)

The presence of CD8^+^ Cytotoxic T lymphocytes (CTLs) in HCC tissue is associated with improved survival. However, the efficacy of CTLs-mediated anti-tumor immune response is functionally limited through diverse mechanisms. Physical conditions (e.g. overload of lactic acid, low pH, hypoxia) [67], severe “metabolic competition” with tumor cells, a lack of CD4^+^ T cells help (moreover, interact with Tregs and other suppressor cells) [64, 68], and high expression of a large amount of immunoregulatory molecules in T cells or HCC cells (e.g. IL-10, Fas/FasL, CXCL17, VEGF, indoleamine-2,3-dioxygenase and so on) [67, 69–71], may be responsible for restricted tumor-associated antigens (TAAs)-specific CD8^+^ T cell responses and poor IFN-γ production of CTLs [72, 73]. Apart from the classic immunosuppressive cells in TME, other components critically manipulate the functions of CTLs as well. Liver fibrosis, a prominent characteristic of HCC, impairs platelet-derived CD44 recognition by CD8^+^ T cells, reducing effector CD8^+^ T cells infiltration, and adhering to liver sinusoids to perform immunosurveillance [74]. Expression of Fas/FasL in CD8^+^ T cells positively correlates with HCC anti-tumor immunity [69]. Recent evidence indicates that tumor-derived vascular endothelial growth factor A (VEGF-A) and prostaglandin E2 (PGE2) cooperatively induce FasL expression in endothelial cells, which leads to excessive turnover of CD8 + T cells and reduce anti-tumor immune responses [71]. CD14^+^ dendritic cells (CD14^+^ DCs), a newly discovered immune regulator of HCC, has been suggested to suppress CTLs via IL-10 and indoleamine-2,3-dioxygenase (IDO) production, and the two cytokines play central roles in various physiological and pathological immune responses and inflammatory processes [75].

Notably, immune checkpoint signaling, which involves enhancement of numerous inhibitory co-stimulatory molecules (e.g. PD-1, LAG-3, CTLA-4, Tim-3, 2B4), has been demonstrated to dramatically induce CTLs exhaustion [58, 76, 77]. More details will be discussed in the section “immune checkpoint pathways and related therapeutics”.

### Innate immune players and stromal components

#### Natural killer (NK) cells

Natural killer (NK) cells constitute a large proportion of the innate immune system in the liver. As the first line of host defense against viral infections (e.g. HBV, HCV) and carcinogenesis, NK cells play a key role in maintaining the balance between immune defense and tolerance. Increasing evidence suggests that hypoxic stress in HCC tissue, switch of activating/inhibitory NK receptors (NKRs) and influences by immune regulatory components in TME, largely contribute to NK cells dysfunction, which significantly correlates with fatigue anti-tumor immunity and poor prognosis [78, 79].

α-Fetoprotein (AFP) overexpressed by HCC cells was demonstrated to indirectly impair interlukine-12 (IL-12) production from dendritic cells (DCs), which results in attenuated cytotoxic effector molecules release, decreased expression of natural killer group 2, member D (NKG2D), an activating receptor on NK cells, and subsequently inhibiting activation and ability of NK cells [80, 81]. A recent study also indicates AFP may exert dual effects on NK cells functions in a direct manner. Short-term exposure to AFP induces IL-2 hyperresponsive phenotype NK cells, accompanied with elevated secretion of IL-1β, IL-6 and TNF-α [82]. These pro-inflammatory cytokines were associated with a low recurrence rate and a prolonged overall survival (OS) of HBV-related HCC patients [83]. In contrast, extended effect of AFP negatively affects long-term NK cell viability [82].

**Fig. 2:**
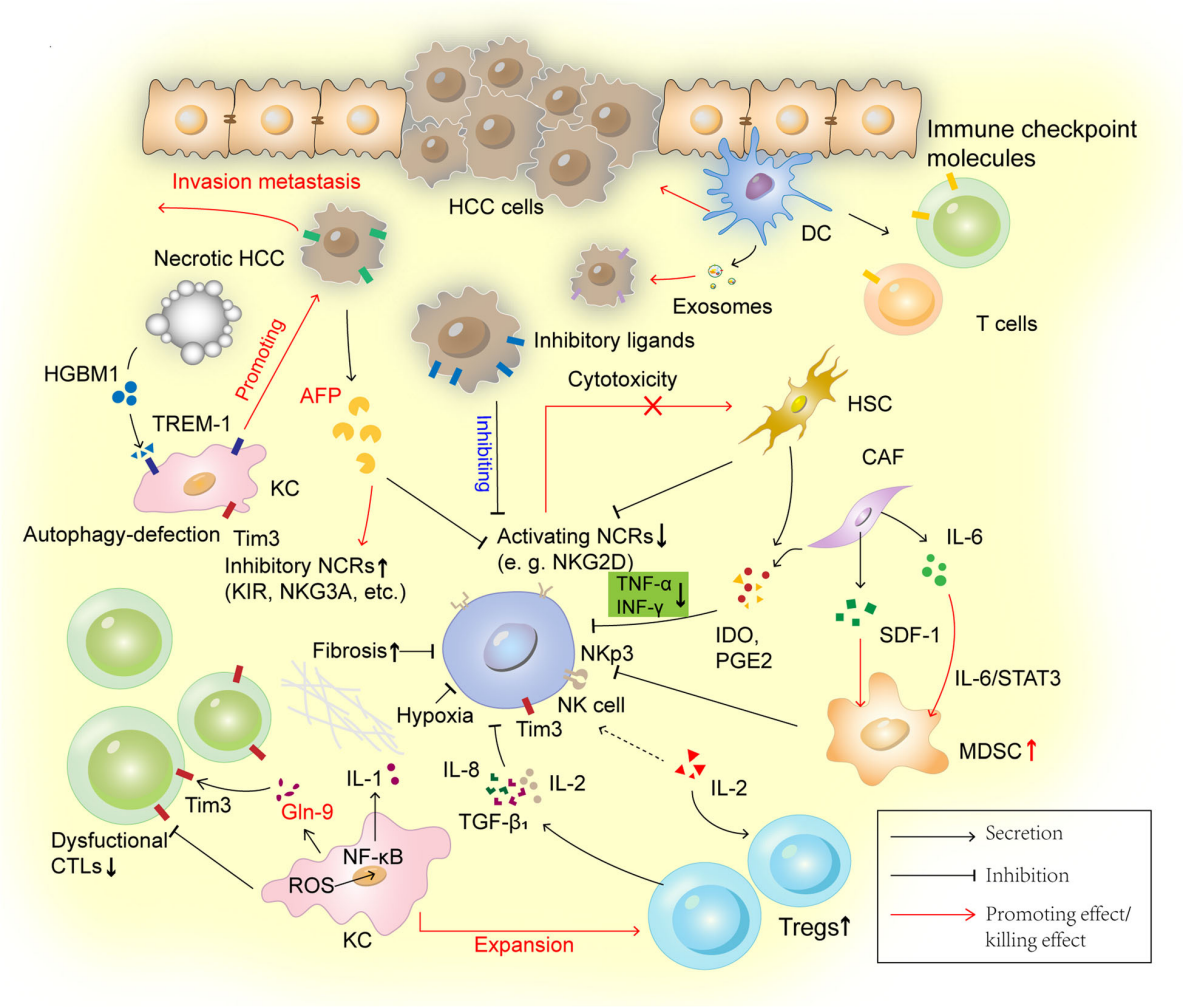
Modulator role of NK cells in regulating HCC immune responses. NK cells exert multiple immune regulatory functions in HCC. Apart from the direct influences on tumor cells, interactions between NK cells and other immune cells or tumor stromal components have been demonstrated to mediate HCC immune evasion

Other modulators in TME of HCC also exert multiple effects on NK activities (Fig. 2**.**). As mentioned above, MDSCs and TAMs infiltration inhibit autologous NK cell cytotoxicity and cytokine production, and the suppression is mainly dependent on NKp30 on NK cells [28]. Tregs compete with NK cells for IL-2 availability and impair NK responses via cytokines release, such as IL-8, TGF-ß1 and IL-10, which then down-regulates expression of NKR ligands on hepatic stellate cells (HSCs) and inhibits their recognition by NKG2D [84]. Hepatocellular carcinoma-associated fibroblasts (CAFs or TAFs), has been shown to induce MDSCs generation through the IL-6/STAT3 axis and stromal cell-derived factor (SDF)-1α secretion [18]. In addition to its direct influence on immunosuppressive TME, CAFs-derived IDO and PGE2 attenuate NK cells-mediated TNF-α and IFN-γ production, which may be associated with persistent fibrosis in HCC and tumor cell immune evasion [85, 86].

#### Kupffer cells (KCs)

KCs have previously been demonstrated to constitute an important part in maintaining liver immune homeostasis. Some studies reported that IL-10-mediated suppression of KC-derived inflammatory TNF-α and NO production contribute to attenuation of hepatitis [87]. Although KCs were once regarded as a powerful line of defense against tumors in the liver, recently, KCs have commonly been explored as pro-carcinogenic stakeholders in the context of HCC, more underlying mechanisms about their immune regulator roles, and KCs-related innate or adaptive immune response have been gradually uncovered. Current investigations indicate that altered functions of KCs are mainly influenced by pro-inflammatory signals and other suppressive cells (e.g. MDSCs) [88]. Triggering receptors expressed on myeloid cells-1 (TREM-1) expressed by KCs, is a crucial factor in HCC initiation. New studies suggest that the potential ligand for TREM-1, high mobility group Box 1 (HMGB1) released by necrotic hepatocytes, is likely involved in activating KCs pro-inflammatory signaling and promoting HCC progression [89]. Autophagy-defective KCs, a novel non-parenchymal liver cellular degradation deficiency, has been shown to promote liver fibrosis, inflammation and hepatocarcinogenesis during the pre-neoplastic stage via enhancing the mitochondrial ROS/NF-κB/IL-1 pathway [90]. In addition, KCs-derived galectin-9, the natural ligand for the T cell immunoglobulin domain and mucin domain protein 3 (Tim-3), leads to expansion of CD4^+^ CD25^+^ FoxP3^+^ Tregs, contraction of CD4^+^ effector T cells, and apoptosis of CTLs in HCC [91]. Galectin-9 not only mediates T-cell senescence in HBV-associated HCC, significantly contributes to the inflammatory reactions and HCC immune escape [92], but notably also represents a potential biomarker of liver fibrosis and may emerge as a novel immunotherapeutic target for treating HCC and liver viral infections [92, 93].

#### Dendritic cells (DCs) and DC-based vaccines

Similar to NK cells, as another major player of innate immunity, DCs serve as professional antigen-presenting cells that are able to prime T-cells against tumor associated antigens (TAAs) involved in HCC progression. Recently, DCs have been an area of high interest as novel vaccines based on DCs have been developed and widely used in treating solid tumors including prostate cancer, melanoma, renal cancer and HCC [94]. DCs engineered with tumor-associated antigens (TAAs), which have been clarified by numerous in vitro and in vivo studies, are regarded as promising vaccines in HCC immunotherapy. In addition, autologous DCs pulsed ex vivo with the lysate of autologous tumor cells, HepG2 cells and telomerase peptides, have been evaluated in human clinical trials.

Recently, the Dendritic cell (DC)-derived exosomes (DEXs) and tumor cell–derived exosomes (TEXs), which elicit tumor regression in autochthonous HCC mouse models, form a new class of cell-free vaccines and extend options for HCC immunotherapeutic vaccines [95, 96] **(**Table 1**.)**.

**Table 1:** Biological effects of DCs-based vaccines in HCC: representative in vitro and in vivo investigations

Agents	Descriptions	Trial category	Biological effects or clinical results	References
AFP and interleukin 18 engineered DCs (AFP/IL-18-DCs)	DCs co-transduced with the AFP gene and IL-18	In vitro studies	• Significantly increase the production of IFN-γ • Promote CD4^+^ T cells proliferation; elevate CTLs activity against AFP-expressing HCC cells	[97]
DCs pulsed with NY-ESO-1	DCs pulsed with the recombinant NY-ESO-1 protein	In vitro studies	• Be more effective in stimulating T cell proliferation compared with immature DCs	[98]
IL-12 engineered DCs (IL-12-DCs)	Endogenous IL-12-expression by adenoviral gene transfer effectively enhances immunostimulation of DC	Translational trials with murine models	• Induce a sufficient Th1 TME allowing the recruitment of T_eff_ to enhance anti-tumor immunity • Improve dendritic cells (DCs)-based immunotherapy of HCC	[99]
CD40 Ligand-Expressing DCs	Transduction of TAA-pulsed DCs with CD40L-encoding adenovirus (Ad-CD40L)	Translational trials with mice models	• Promote DC immunostimulation with up-regulation of CD80/CD86 and IL-12 expression • Increase tumor infiltration with CD4^+^, CD8^+^ T cells and NK cells • Elevate IFN-γ release and CTLs cytotoxicity	[100]
TEXs pulsed DCs	Tumor cell derived exosomes (TEXs)-pulsed DCs	In vitro and in vivo orthotopic *HCC* mice models	• Increase numbers of T lymphocytes infiltration, elevate IFN-γ production; decrease IL-10, TGF-β in tumor sites • Elicit a stronger immune response than cell lysates in vitro and in vivo	[95]
A new form vaccine: DCs-DEXs	Exosomes derived from AFP- expressing DCs	Translational investigation in mouse models	• A cell-free vaccine option for HCC immunotherapy • Decrease Tregs infiltration, IL-10, TGF-β in tumor sites • Reshape the TME in HCC	[96]
TAAs pulsed DCs vaccine	α-fetoprotein, glypican-3 and MAGE-1 recombinant fusion proteins pulsed DCs	A prospective phase I/II clinical study in 5 HCC patients	• Result: safe and well-tolerated • Over 95% of DCs demonstrated highly expressed MHC class I (HLA-ABC), MHC class II (HLA-DR), and costimulatory molecules (CD86, CD80, and CD40) • Induce Th1 immune responses with highly produced IL-12, IFN-γ • Trigger stronger CTLs responses	[101]
TAAs pulsed DCs vaccine	α-fetoprotein, glypican-3 and MAGE-1 recombinant fusion proteins pulsed DCs	A prospective phase I/II clinical study in 12 HCC patients	• Result: safe and well-tolerated • 1-, 2-, and 5-year cumulative RFS rates were improved	[102]
DCs pulsed with tumor cell lysate	Mature autologous DCs pulsed exvivo with HepG2 lysate	A phase II clinical trial with 35 patients with advanced HCC	• Result: safe and well-tolerated • MS: 168 days; 6-month survival rate: 33%; 1-year survival rate 11% • Induce stronger T cell responses and IFN-γ release	[103]
DCs pulsed with tumor cell lysate	Mature autologous DCs pulsed ex vivo with HepG2 lysate	A clinical trial with 2 groups: Group1: 15 advanced HCC patients received DCs vaccination Group2: control group	• Result: safe and well-tolerated • CD8^+^ T cells and serum IFN-γ were elevated after DCs injection • Partial radiological response: 13.3%; stable course: 60%; and 26.7% showed progressive disease and died at 4 months post-injection	[104]
DCs pulsed with AFP	AFP peptides pulsed onto autologous DCs	A phase I/II clinical trial in which HLA-A*0201 patients with AFP-positive HCC, 10 patients received DCs vaccination	• 6 of 10 subjects increased IFN-γ producing AFP-specific T cell responses	[105]

Notes: *TAA* tumor-associated antigens, *MAGE-1* melanoma-associated antigen 1, *GPC-3* glypican-3, *IL-12* interleukin-12, *AFP* a-fetoprotein, *TEXs* tumor cell–derived exosomes, *TGF-β* transforming growth factor-β, *TME* tumor microenvironment, *IFN-γ* interferon-γ, *DEXs* dendritic cell-derived exosomes, *CTLs* cytotoxic T lymphocytes, *Tregs* regulatory T cells

### Representative immune inhibitory factors and modulators

The abundance of pro-inflammatory chemokines, cytokines and immunosuppressive molecules, which orchestrates a strongly immunosuppressive tumor milieu, play critical roles in reshaping TME, mediating intercellular crosstalk, and exerting immune evasion-promoting effects of HCC. Some of their specific functions have been mentioned while discussing immune cells of HCC, here, we summarize the representative players that current studies mainly highlight (Table 2**.**).

**Table 2:** Representative molecules and signaling pathways mediated pro−/anti-tumor immunity of HCC

Cytokines/signaling molecules	Category	Description	References
IL-1β	Pro-inflammatory cytokine	• A favorable factor for prolonged OS of HBV-related HCC patients • TAMs-secreted IL-1β in HCC contributes to HIF-1α stability, IL-1β/HIF-1α induce EMT and metastasis of HCC	[18] [37]
IL-12	Pro-inflammatory cytokine (anti-tumor immunity modulator)	• Promote cytotoxicity and IFN-γ production • Mediate CD4^+^ T helper cells transformation to Th1 phenotype, enhance cell based immunity • Up-regulate NKG2D related NKs anti-tumor immunity	[81–83]
IL-8	Pro-inflammatory cytokine	• Trigger potent pro-inflammatory signals in HCC; promote HCC immune evasion and metastasis • Enhance HCC-related fibrosis and Tregs enrichment in tumor tissue	[33, 84, 106]
IL-10	Inhibitory cytokine that involves in both innate and adaptive immunity in HCC	• Tolerogenic DCs/ FcγRII^low/−^B cells derived IL-10 induces hepatic tolerance by promoting T cell hypo-responsiveness • Suppress CD4^+^ T cells activity via CTLA-4-dependent manner • IL-10 production is associated with Foxp3^+^ Tregs accumulation in HCC • Accelerate HCC progression by mediating polarization of alternatively activated M2 macrophages	[38, 39, 75] [107, 108]
IL-6/STAT3	Pro-inflammatory/carcinogenesis signaling	• Mediate MDSCs activation then result in immunosuppression • Up-regulate IL-10, IDO expression; down-regulate IFN-γ; induce T cells dysfunction and apoptosis	[18, 109]
PD-1/PD-L1	Immune checkpoint molecules	• Impairing anti-tumor immunity and promotes CD8^+^ T cells exhaustion and apoptosis • PD-1 over-expressed myeloid cells, such as DCs, suppress T cell responses in HCC	[110, 111]
LAG3	Immune checkpoint molecule	• Up-regulated on TAA-specific T cells • Significantly impairs CD4^+^ and CD8^+^ TILs functions in HCC	[112]
CTLA-4	Immune checkpoint molecule	• Mediates immunosuppression by inducing Tregs activity and IDO and IL-10 productions in DCs • Suppresses the proliferation of T cells	[73, 112]
Tim3/Galectin-9 pathway	Immune checkpoint signaling	• Negatively regulates Th1-mediated immune responses • Mediates CTLs dysfunction and immunosuppressive responses in HBV-associated HCC • Fosters HCC development by enhancing TGF-β-mediated alternative activation of macrophages	[27, 43, 76, 113]
VEGF, PDGF, HGF	Major growth factors in TME of HCC	• Enhance interactions between TAFs/HSCs and HCC cells • Mediates recruitment of immune inhibitory cells • Mediates other pro-inflammatory signals in TME (e.g. IL-6/STAT3 axis) • Promotes angiogenesis and immune evasion	[18, 75]
IDO	Immunosuppressive modulator	• High level IDO expression is associated with poor prognosis and high recurrence rate in HCC patients; a potential target for HCC immunotherapy • Enhance regulation of immune responses, such as T-cell proliferation impairment, promotion of Tregs expansion • IDO derived from HSCs and CAFs impair cytotoxicity and cytokine production of NK cells • CD14^+^CTLA-4^+^ regulatory DCs derived IDO suppress CTLs response; cause NKs dysfunction in HCC anti-tumor immunity	[75] [83] [85, 109] [114]
SDF-1α/CXCR4	A multiple signaling that mediates HCC immune evasion, progression and metastasis	• Enhance interactions between TAFs/HSCs and HCC cells • Facilitate MDSCs recruitment and generation, then results in immune evasion • Contribute to HCC fibrosis and hypoxia • Synergize with other stroma-derived cytokines (such as HGF, VEGF, TGF-β and so on), promoting HCC growth, angiogenesis, metastasis	[18, 115] [116]
CXCL17	119-amino acid chemokine	• An independent factor that correlates with HCC regulatory immune cells infiltration • Predict poor prognosis of HCC	[70]
CCL2(also named MCP-1)	Multifunctional factor	• Multiple cellular resources, including HSCs, hepatocytes, macrophages and so on • CCL2/CCR2 promotes regulatory cytokines release, M2-macrophages accumulation and polarization • Suppress cytotoxic CD8+ T lymphocytes anti-tumor responses • Facilitate TANs infiltration in HCC	[54, 117] [118]
Hypoxia (HIF-1α)	Versatile modulator of TME and tumor immunotolerant state	• Promote recruitment of Treg, MDSCs. • regulate release of multiple chemokines and inflammatory factors; Activate transcription of C-C motif ligand 26, 28 (CCL26, CCL28) and interleukines (ILs). • contribute to immune tolerance and angiogenesis.	[19, 119, 120]
CXCL1/CXCR2 signaling	Immunosuppressive signaling axis	• Impair immune balance in TME of HCC. • Facilitate immune escape via increasing MDSCs recruitment and repressing infiltration of IFNγ+CD8+ T cells.	[121]
CXCL5	C-X-C motif chemokine	• Recruits more TANs infiltration and contributes to TANs-induced HCC immune evasion.	[55]
CCL15	Immunosuppressive signaling	• Serves as an independent factor for HCC prognosis and survival. • Recruit CCR1 + CD14+ monocytes infiltration, accelerate tumor proliferation and metastasis by activating STAT1/erk1/2 signaling. • Upregulate immune checkpoints (e.g. PD-L1, Tim3) and immune tolerogenic enzymes (e.g. IDO, ARG)	[122]

Notes: *HCC* hepatocellular carcinoma, *IL-* interleukin-, *OS* overall survival, *EMT* epithelial-mesenchymal transition, *HIF-1α* hypoxia inducible factor-1, *IFN-γ* interferon-γ, *NKs* natural killer cells, *Tregs* regulatory T cells, *DCs* dendritic cells, *MDSCs* myeloid-derived suppressor cells, *PD-1* programmed cell death protein 1, *PD-L1* programmed death-ligand 1, *LAG3* lymphocyte-activation gene 3, *TAA* tumor associated antigen, *TILs* tumor infiltrating lymphocytes, *CTLA-4* cytotoxic T-lymphocyte-associated protein 4, *IDO* indoleamine 2,3-dioxygenase, *Tim3* T cell immunoglobulin mucin, *CTLs* cytotoxic T lymphocytes, *VEGF* vascular endothelial growth factor, *PDGF* platelet-derived growth factor, *HGF* hepatocyte growth factor, *TME* tumor microenvironment, *TAFs* tumor-associated-fibroblasts, *HSCs* hepatic stellate cells, *CAFs* cancer associated fibroblasts, *SDF-1α* stromal cell derived factor 1α, *CXCR4* chemokine (C-X-C motif) receptor 4, *CXCL17* chemokine (C-X-C motif) ligand 17, *CCL2* chemokine (C-C motif) ligand 2, *MCP-1* monocyte chemotactic protein 1, *TANs* tumor-associated neutrophils**,** *CXCL1* chemokine (C-X-C motif) ligand 1**,** *CXCR2* chemokine (C-X-C motif) receptor **2,** *CXCL5* chemokine (C-X-C motif) ligand **5,** *CCL15* chemokine (C-C motif) ligand **15,** *CCR1* chemokine (C-C motif) receptor **1,** ***ARG*** Arginase

### Current immunotherapeutic strategies for HCC

**Fig. 3:**
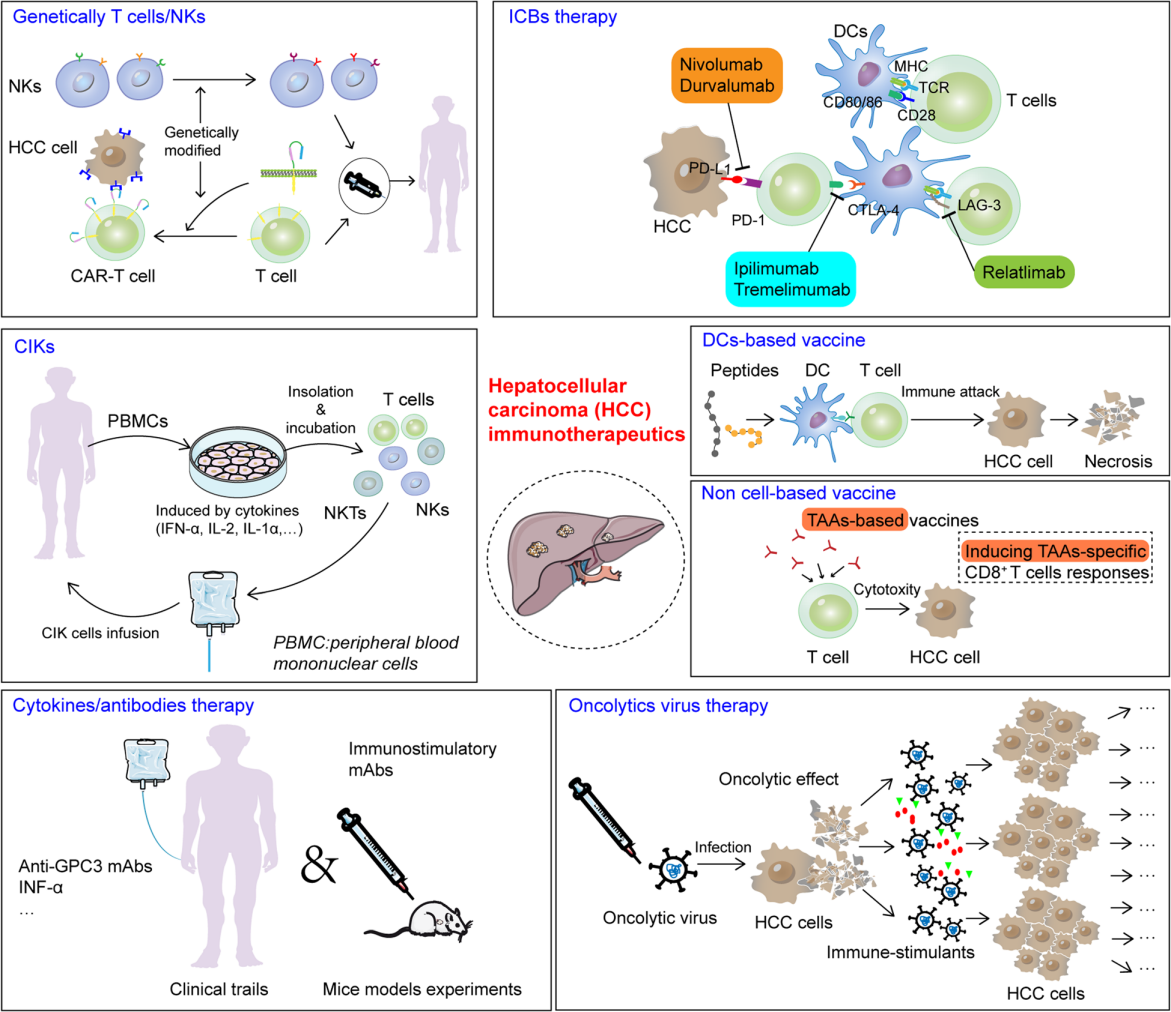
Current immunotherapeutic options for HCC. Immunotherapeutic approaches for HCC mainly include immune-checkpoint blockade (ICB), cell-based (mainly refers to DCs) /non-cell based vaccines, adoptive cell transfer (ACT), cytokine/antibody based immune regimens and oncolytic virus

As an inflammation-associated cancer, HCC represents a promising target for immune based therapeutics. Clinically, the success of immune oncology in many types of cancer has encouraged implementation of immunotherapeutics in HCC. Recent studies have suggested that tumor antigen-specific immunotherapy and other approaches modulating immunogenicity have become attractive strategies for HCC treatment. Generally, these immunotherapeutic approaches for HCC could be mainly categorized into immune-checkpoint blockade (ICB), cell-based (mainly refers to DCs) /non-cell based vaccines, adoptive cell transfer (ACT), cytokine/antibody based immune regimens, and combination of immunotherapeutic agents with other drugs (Fig. 3**.**). Here, we collect some representative data on preclinical and clinical trials on immune based strategies of HCC, and discuss our current knowledge on their action mechanisms, rationale and application prospects for HCC treatment in the foreseeable future.

### Immune checkpoint inhibitors

Immune checkpoints are a specific sub-type of membrane-bound molecules that act as pivotal regulators of immune escape in cancers. The most studied immune checkpoints in HCC includes cytotoxic T lymphocyte protein 4 (CTLA-4), programmed cell death protein-1 and its ligand (PD-1, PD-L1), lymphocyte activation gene 3 protein (LAG-3) and mucin domain-containing molecule-3 (Tim-3).

#### Programmed cell death protein-1 and its ligand (PD-1, PD-L1)

PD-1, a regulator immunoglobulin expressed on activated CD4^+^, CD8^+^ T cells, B cells and NK cells, plays an important role in maintaining immune tolerance and repressing cytotoxicity of T lymphocytes [123]. Co-inhibitory signals in lymphocytes are mediated by binding of PD-1 to its ligands PD-L1 (B7-H1) and PD-L2 (B7-DC) [124]. In HCC, it’s clear that an increase in the number of both circulating and intratumoral PD-1^+^ CD8^+^ T cells predict high postoperative recurrences and poorer prognosis. It is also known that up-regulation of PD-L1 on HCC cells, which is induced by various cytokines, particularly IFN-γ, in turn contributes to impairing anti-tumor immunity and promotes CD8^+^ T cells apoptosis [110]. New in vitro and in vivo discoveries indicate that PD-1 overexpressed myeloid cells, such as DCs, suppress T cell responses in HCC. CD8^+^ T cells can be more potently activated to secrete IL-2 and IFN-γ via adoptive transfer of PD-1-deficient DCs [111].

Clinically, a representative phase 1/2 dose escalation and expansion trial on PD-1 immune checkpoint inhibitors Nivolumab (CheckMate 040 study) showed a promising role for immunotherapy in the treatment of advanced HCC, and relevant results were presented at the 2017 ASCO annual meeting [125]. In dose-escalation phase (enrolled number = 48), the objective response rate (ORR) was 15%, the disease control rate (DCR) was 58% and the median time to progression was 3.4 months. In dose-expansion phase (total number = 214; in 4 cohorts), generally, the ORR was reported as 20%, the DCR was 64%, the median time to progression was 4.1 months, and the 6-month and 9-month progression-free survival rates were 83 and 74% respectively. A subsequent CheckMate-040 based analysis compared the ORR and survival between intent-to-treat (ITT) overall population and Asian cohort. It suggested that Nivolumab showed similar mOS and manageable safety profile both in ITT population and Asian patients [126].

The efficacy of another anti-PD-1 monoclonal antibody, Pembrolizumab, was assessed in a phase II, open-label trial (KEYNOTE-224). In this study, Pembrolizumab was proven to be effective and well-tolerated in Sorafenib-experienced patients with advanced HCC, and PD-L1 expression level may act as a useful predictive bio-marker in selecting interested HCC patients. A total of 104 enrolled patients in this study represented 8-month median duration of response (mDOR), with median time to response of 2 months [127].

In addition, another phase 3 randomised clinical trial of nivolumab mono-therapy compared with sorafenib in the first-line setting is ongoing (NCT02576509). Moreover, combination therapies of anti-PD-L1 antibody (Duvalumab) with anti-CTL4–4 antibody (Tremelimumab) for unresectable HCC are under study as well (NCT02519348).

Other reported combination immunotherapy studies are encouraging and really open new avenues for HCC treatment [128–130], however, additional strategies are needed to uncover more sensitive predictive biomarkers besides PD-1/PD-L1 axis, optimize treatment selection, and improve HCC patients immune response. (More data of terminated or ongoing clinical trials are available in Table 3**.**).

**Table 3:** Representative ongoing immune checkpoint blockade(ICB) based immunotherapy clinical trails in HCC

Regimen	Disease	Mechanism of action	Estimated/Actual enrollment	NCT number
Anti-CTLA-4 antibody based monotherapy/combination therapy				
Tremelimumab+TACE	Liver cancer	Anti-CTLA-4 antibody; chemoembolization	61	NCT01853618
Tremelimumab	Advanced HCC	Anti-CTLA-4 antibody	20	NCT01008358
Ipilimumab +Nivolumab/ Nivolumab alone following SBRT	Unresectable HCC	Anti-PD-1 antibody, anti-CTLA-4 antibody	50	NCT03203304
Anti-PD-1 antibody based monotherapy/combination therapy				
Nivolumab+Y90 Radioembolization	HCC	Liver-localized radioembolization, PD-1 blockade	40	NCT03033446
Nivolumab+cabozantinib	Advanced HCC	Neoadjuvant therapy, PD-1 blockade	15	NCT03299946
Nivolumab+Pexa Vec	HCC	Oncolytic Immunotherapy, PD-1 blockade	30	NCT03071094
Nivolumab+Ipilimumab	HCC (Resectable and potentially resectable)	CTLA-4 blocade, PD-1 blockade	45	NCT03222076
Nivolumab following selective internal radiation therapy (SIRT)	HCC (unresectable)	PD-1 blockade, radiation therapy	40	NCT03380130
Nivolumab following complete resection	HCC	PD-1 blockade	530	NCT03383458
Nivolumab+Galunisertib	NSCLC HCC	TGF-β receptor I kinase inhibitor, PD-1 blockade	75	NCT02423343
Nivolumab+Lenvatinib	HCC	TKI + PD-1 blockade	26	NCT03418922
Nivolumab+Y90	HCC	PD-1 blockade+Radioembolization	35	NCT02837029
Nivolumab+Sorafenib	HCC	PD-1 blockade+chemotherapy	40	NCT03439891
Nivolumab+CC-122 (Avadomide**)**	HCC (unresectable)	PD-1 blockade+immunomodulator (targeting protein cereblon)	50	NCT02859324
Nivolumab+deb-TACE	Advanced HCC	PD-1 blockade+transarterial chemoembolization	14	NCT03143270
Nivolumab+Mogamulizumab	HCC other solid tumors	PD-1 blockade+anti-CCR4 antibody	188	NCT02705105
TATE followed by Nivolumab or Pembrolizumab	HCC; mCRC	PD-1 blockade+TACE	40	NCT03259867
Nivolumab	Advanced HCC (with or without viral infections)	PD-1 blockade	262	NCT01658878
Nivolumab (vs. Sorafenib)	Advanced HCC	PD-1 blockade	726	NCT02576509
Anti-PD-L1 antibody based monotherapy/combined therapy				
Durvalumab+tremelimumab	Unresectable HCC	Anti-PD-L1 antibody, anti-CTLA-4 antibody	440	NCT02519348
Durvalumab monotherapy; Durvalumab+Tremelimumab vs. Sorafenib	Unresectable HCC	Anti-PD-L1 antibody, anti-CTLA-4 antibody	1200	NCT03298451
Durvalumab+Guadecitabine (SGI-110)	Liver cancer; pancreatic cancer; bile duct cancer; gallbladder cancer	Anti-PD-L1 antibody, small molecule DNA methyltransferase 1 (DNMT1) inhibitor	90	NCT03257761
Durvalumab+Tremelimumab+ablative therapies	Advanced HCC and BTC	Anti-PD-L1 antibody, anti-CTLA-4 antibody	90	NCT02821754
Durvalumab+Ramucirumab (LY3009806)	GEJ adenocarcinoma; NSCLC; HCC	Anti-PD-L1 antibody, anti-VEGFR2 antibody	114	NCT02572687
Anti-LAG-3 antibody in combination with anti-PD-1 blockade				
Relatlimab+Nivolumab	Different types of solid tumor (including HCC)	Anti-LAG-3 antibody,anti-PD-1 antibody	1000	NCT01968109

Notes: *Y90* yttrium Y 90 glass microspheres, *deb-TACE* drug eluting bead transarterial chemoembolization, *TATE* transarterial tirapazamine embolization, *mCRC* metastatic colorectal cancer, *BTC* biliary tract carcinomas, *GEJ* gastroesophageal junction, *SBRT* stereotactic body radiotherapy

#### Cytotoxic T lymphocyte protein 4 (CTLA-4)

Cytotoxic T lymphocyte protein 4 (CTLA-4), an inhibitory co-receptor that is expressed by activated T cells and is constitutively present on Tregs, has great affinity for competing with CD28 by binding to its ligands, CD80 and CD86, on antigen presenting cells (APCs). CTLA-4 plays a critical part in controlling CD4^+^ T cells function. In HCC and many other types of cancer, CTLA-4 suppresses the proliferation of T cells that have undergone TAA recognition and differentiation [131]. Additionally, inside HCC tissues, CTLA-4 further mediates immunosuppression by inducing Tregs activity and IDO and IL-10 productions in DCs [75].

Many clinical trials of antibodies targeting CTLA-4 are ongoing. A pilot clinical investigation testing anti-tumor and anti-viral effects of Tremelimumab in patients with HCC and HCV infection showed strong signs of anti-tumour efficacy (NCT01008358). The treatment presents a reliable safety profile, as no immune-related adverse events occurred. The median time to progression (TTP) was 6.48 months, and median overall survival (OS) was 8.2 months. The partial response rate was observed as 17.6%, and had a remarkable disease control rate of 76.4%. Moreover, 36% of the patients with AFP levels > 100 ng/ml showed more than 50% drop after Tremelimumab therapies [132]. In another phase 1 clinical study that tests the safety and effectiveness of Tremelimumab with radiofrequency (RFA) (NCT01853618), the median TTP and median OS were respectively 7.4 months (95% CI 4.7 to 19.4 months) and 12.3 months (95% CI 9.3 to 15.4 months). The 6-week HCC biopsies showed a clear increase in CD8^+^ T cells infiltration demonstrating that the combination of Tremelimumab with RFA in advanced HCC is feasible and results in stronger anti-tumor immunity [133].

#### Mucin domain-containing molecule-3 (Tim-3) and lymphocyte activation gene 3 protein (LAG-3)

Mucin domain-containing molecule-3 (Tim-3) is a transmembrane protein that is expressed on IFN-γ-secreting Th1 cells, NK cells and CTLs [113]. Tim-3 interacts with its soluble ligand galectin-9, and then negatively regulates T cell responses [91]. The expression of Tim-3 is increased in T cells infiltrating in chronic HBV infection [134], and the Tim-3/galectin-9 pathway consistently predicts poor prognosis in patients with HBV-associated HCC [76].

Lymphocyte activation gene 3 protein (LAG-3), a member of the immunoglobulin super-family proteins, which often binds MHC class II molecules with high affinity, represses the co-stimulatory functions of T cells [135]. Clinically, dual blockade of LAG-3 with anti-PD-1 therapy is being tested in a Phase I trial (NCT01968109) (Table 3**.**).

The immunosuppressive roles of both Tim-3 and LAG-3 in chronic viral hepatitis and HCC have been uncovered recently. However, their clinical values need to be further elucidated.

### Adoptive cell transfer (ACT) based therapy in HCC

Besides the immune-checkpoint blockade (ICB), other effective immunotherapeutic options for HCC are urgently needed. In recent years, the exploration and development of cell-based immunotherapies in treating solid tumors have received considerable attentions. Adoptive cell transfer (ACT) offers robust and more durable anti-tumor immunity in cancer treatment. Recent translational research and clinical cases reported the success of engineered autologous HBV-specific T cell receptor (TCR) redirected therapeutics in treating HBV-associated HCC [136, 137], which broadens the immunotherapeutic approaches and might be used to treat a wider population of patients [138]. Based on the cell types, ACT used in HCC pre-clinical/clinical researches can be mainly classified as: (1) cytokine-induced killer (CIK) cells treatment, and (2) genetically modified NK cells or T cells (CAR-T).

CIK cells are a mixture of T lymphocytes, which are ex vivo expanded in the presence of cytokines (such as IL-1, IL-2, IFN-γ), comprising activated NKT cells, CD3^−^/CD56^+^ NK cells, and CD3^+^ /CD56^−^ cytotoxic T cells [139]. CIK cells can be obtained in great numbers from peripheral blood mononuclear cells (PBMCs) and are very easily cultured. More importantly, it has been clarified that the absence of MHC restrictions favors CIK cells’ more potent anti-tumor efficacy compared with traditional CTLs [58, 139]. Previous studies suggested that CIK cells prevent HCC from progression, and effectively kill cancer stem cells (CSCs) mainly through NKG2d-ligands recognition [140]. A retrospective study demonstrated a significant correlation between high number of PD-1^+^ TILs and favorable outcome in CIK cells treated HCC group, which suggested that PD-1^+^ TILs may be utilized to predict the efficacy of CIK treatment in post-operative HCC patients [141]. A randomized, phase 3 clinical trial of the efficacy and safety of CIK cells treatment in 230 patients with HCC (NCT00699816) indicates that for post-curative treatment in HCC patients, adjuvant immunotherapy with CIK cells can prolong recurrence-free survival and OS (median RFS:44.0 months in treatment group, 30.0 months in the control group). Additionally, the proportion of patients with serious adverse events did not differ significantly between the treatment and control groups [142]. Several trials of CIK cells treatment in combination with other therapies, such as RFA, arterial chemoembolization and epitope-pulsed DCs, have been reported [143–145]. The evidence obtained from a growing body of literature confirms that CIK cells is a very promising adoptive immunotherapy that can be exploited for treatment and prevention of recurrence in HCC. However, a small fraction of patients undergoing standard therapies suffer from ‘immune fatigue’ status, and lack adequate leukocytes [139], a major obstacle for CIK cells treatment that needs to be overcome urgently. (Ongoing CIK-based clinical trials are available in Table 4**.**)

**Table 4:** Clinical trials based on CIKs and genetically modified T cells under study for the treatment of HCC

Regimen	Population	Design	Estimated/Actual enrollment	NCT number
CIKs mono-therapy for HCC				
CIKs	• Hepatocellular carcinoma	• Phase 3 clinical trial • CIK treatments within 3 months after liver resection	200	NCT00769106
CIKs	• Hepatocellular carcinoma • Renal cell carcinoma • Lung cancer	• Phase 1 clinical trial • CIK treatments following radical resection	40	NCT01914263
CIKs	• Hepatocellular carcinoma	• Phase 3 clinical trial • CIK treatments following radical resection	200	NCT01749865
DC-CIKs	• Hepatocellular carcinoma	• Phase 2 clinical trial • Dendritic and CIKs used to treat HCC patients who got CR or PR after complete resection/ TACE	100	NCT01821482
CIKs in combination with other therapies for HCC				
CIKs+ anti PD-1 antibodies	• Hepatocellular carcinoma • Renal cell carcinoma • Bladder cancer • Colorectal cancer • Non-small-cell lung cancer • Breast cancer	• Phase 2 clinical trial • Combination therapy	50	NCT02886897
CIKs+ TACE	• Hepatocellular carcinoma • Digestive system neoplasms	• Phase 3 clinical trial • Combination therapy	60	NCT02487017
CIKs+ RFA	• Hepatocellular carcinoma	• Phase 3 clinical trial • RFA + Highly-purified CTL vs. RFA Alone for Recurrent HCC after partial hepatectomy	210	NCT02678013
CAR-T trials for HCC treatment				
Anti-GPC3 CAR-T	• Hepatocellular carcinoma (GPC3 + advanced HCC)	• Phase 1/2 clinical trial	20	NCT03084380
Anti-GPC3 CAR-T	• Hepatocellular carcinoma (GPC3 + advanced HCC)	• Phase 1/2 clinical trial	60	NCT02723942
Autologous anti-AFP (ET1402L1)-CAR-T	• AFP expressing hepatocellular carcinoma	• Phase 1 clinical trial • The second generation CAR-T treatment	18	NCT03349255
Anti-GPC3 CAR-T	• Advanced hepatocellular carcinoma	• Phase 1 clinical trial	13	NCT02395250
Anti-GPC3 CAR-T	• Advanced hepatocellular carcinoma	• Phase 1 clinical trial	30	NCT03198546
TAI-GPC3-CAR-T	• Hepatocellular carcinoma	• Phase 1/2 clinical trial • GPC3-CAR-Ttreatment mediated by the method of transcatheter arterial infusion (TAI)	30	NCT02715362
Anti-GPC3 CAR-T	• Advanced hepatocellular carcinoma	• Phase 1/2 clinical trial • GPC3-CAR-Ttreatment by intratumor injection	10	NCT03130712
Anti-Mucin1 (MUC1) CAR-T	• Hepatocellular carcinoma • Non-small cell lung cancer • Pancreatic carcinoma • Triple-negative invasive breast carcinoma	• Phase 1/2 clinical trial • Patients with MUC1+ advanced refractory solid tumor	20	NCT02587689
Anti-GPC3 CAR-T	• Relapsed or refractory hepatocellular carcinoma	• A single arm, open-label pilot study • GPC3+ hepatocellular carcinoma	20	NCT03146234
Anti-EpCAM CAR-T	• Colon cancer • Esophageal carcinoma • Pancreatic cancer • Prostate cancer • Gastric cancer • Hepatic carcinoma	• Phase 1/2 clinical trial • Targeting patients with EpCAM+ cancer	60	NCT03013712
CAR-T targeting TAAs	• Hepatocellular carcinoma • Pancreatic cancer • Colorectal cancer	• Phase 1/2 clinical trial • CAR-T targets: GPC3 for hepatocellular carcinoma • Mesothelin for pancreatic cancer • CEA for colorectal cancer	20	NCT02959151

Notes: *TACE* transcatheter arterial chemoembolization, *RFA* radiofrequency ablation, *DC-CIKs* dendritic and cytokine-induced killer cells, *CR* complete remission, *CAR-T cells* chimeric antigen receptor-T cells, *TAI* transcatheter arterial infusion

Another adoptive cell immunotherapy, which uses chimeric antigen receptor-modified T cells (CAR-T) or genetically modified NK cells, has been shown to be a promising strategy for the treatment of HCC. CAR-T can specifically recognize tumor-associated antigens and effectively eliminate tumor cells in a non-MHC restricted manner. Furthermore, additional genes could be used to enable resistance to immune suppression [146]. Successful use of CAR-T cells in treating haematological malignances includes ACT using CD19-specific CAR-T cells and the third-generation of CD20-specific CAR with CD28 and 4-1BB co-stimulatory domains. CAR-T cell treatment is well tolerated and can induce great remission in B-cell lymphoma [147, 148] (NCT00924326, NCT00621452), however, despite this, the achievements of CAR-T cells therapies against solid tumors are still modest. In HCC, recent studies revealed that glypican-3 (GPC-3), an oncofetal proteoglycan anchored to HCC cell membrane that functions to promote HCC progression and is associated with poor prognosis, provides a novel prognostic molecule therapeutic target in HCC immunotherapy [149]. Previous in vitro and orthotopic xenograft models of human HCC experiments both indicated that cytotoxic activities of T cells redirected to GPC-3 seemed to be positively correlated with GPC-3 expression levels in the targeting cell. This suggests that GPC-3-targeted CAR-T cells may offer a promising immune therapeutic intervention for GPC-3^+^ HCC [150, 151]. Moreover, a series of clinical studies conducted to test the safety and efficacy of CAR-T cells redirected to GPC-3 in HCC treatment are underway (Table 4**.**). Similarly, CAR-modified NKs represents a newly emerging immunotherapeutic modality for HCC therapy. Potent anti-tumor responses of GPC-3-specific NKs based therapeutics were observed in HCC xenografts with both high and low GPC-3 expression, which extends treatment options for patients with GPC-3^+^ HCC [152]. Another gene-modified NKs candidate, human interleukin-15 (hIL-15) gene-modified NKL cells (NKL-IL15), has been demonstrated to express high levels of cytolysis-related molecules (TNF-α, IFN-γ, NKp80 and so forth), which induces higher NKG2D ligand expression on target cells and results in increased susceptibility of HCC to NKs-mediated cytolysis [153].

Collectively, genetically modified NK cells and CAR-T cells based treatment provide new avenues for immunotherapies against HCC. Nevertheless, before being widely used as therapeutics in the clinic, their clinical efficacy and on-target-off tumor toxicity still require further assessments in more randomized trials.

### Non-cell based vaccines and oncolytic viruses based immunotherapy in HCC

With the identification of a growing number of tumor-associated antigens (TAAs), and as a result, vaccines targeting HCC TAAs have been investigated and developed. A number of tumor antigens, such as human alpha-fetoprotein (AFP), GPC-3 and telomerase-reverse transcriptase (hTERT) have been identified as vaccine-based immunotherapeutic targets for HCC [154]. (Table 5**.**). Although increased efforts are being made to advance TAAs-based vaccines, the early clinical trials witnessed a mixed history of success and failure [155–157, 160]. The first HCC AFP-vaccine clinical trial was completed with only transient immunological responses detected, partially due to the limited number of antigens used or deficient CD4^+^ helper T cell support [157, 160, 161]. A vaccine with a single 16 amino acids sequence, hTERT-derived peptide (GV1001), and binds multiple HLA class II molecules, results in little clinical activity and no detected absolute antigen-specific CTLs responses [155]. On the other hand, partial clinical data on GPC-3 based vaccines demonstrated that the vaccine could induce measurable anti-tumor responses and are associated with prolonged OS of HCC patients [156, 162].

**Table 5:** Several representative clinical trials of non-cell based vaccines and oncolytic virus (OVs) based immunotherapy in HCC

Trial (the 1st author/ responsible party)	Agent	Design	Population	Status/Relevant results	Registration no.& Reference order
Non-cell based vaccines					
Greten et al. (2010)	GV1001: a telomerase derived peptide vaccine	• A phase 2 open-label trial; 4-week injections with GM-CSF + GV1001 vaccinations • P:tumor response • S:TTP, TTSP, PFS, OS, safety and immune responses	40 patients with advanced HCC	Status: terminated Results: no relevant toxicity, median OS: 11.5 months, median PFS: 57 days, median TTP: 57 days, TTSP: 11.7 months	[155] NCT00444782
Sawada et al. (2012)	GPC-3-derived peptide vaccine	• A phase 1 Trial • P: safety • S:TTP, OS, immune responses (measured by IFN-γ ELISPOT assay)	33 patients with advanced HCC	Status: terminated Results: well-tolerated, 91% patients were successfully induced with CTLs-mediated responses, median OS: 9.0 months, median TTP: 3.4 months, GPC-3-specific CTL frequency after vaccination correlated with OS	[156] UMIN-CTR000001395
Butterfield et al. (2003)	AFP peptide vaccine	• A pilot Phase 1 clinical trial • In vivo studies testing AFP peptide- vaccine reactive T cells responses	6 patients with HCC	Status: terminated Results: all of the patients generated T-cell responses to most or all of the peptides as measured by direct IFN –γ ELISPOT and MHC class I tetramer assays	[157]
Immunitor LLC et al. (2018)	An oral therapeutic vaccine: hepcortespenlisimut-L (Hepko-V5)	• A phase 3, randomized, placebo-controlled, double-blinded trial • P:changes in serum AFP levels, tumor burden, OS	Estimated enrollment:120 patients with advanced HCC	Status: recruiting Results: none	NCT02232490
Roswell Park Cancer Institute (2016)	Vaccine therapy in treating NY-ESO-1 expressing solid tumors	• A phase 1 clinical trial determines the safety of DC205-NY-ESO-1 vaccine	18 patients with NY-ESO-1 solid tumors, including HCC	Status: completed Results: none	NCT01522820
Butterfield et al. (2013)	AFP+ GM-CSF Plasmid Prime and AFP Adenoviral vector Boost	• A phase 1/2 trial • Testing immunization with AFP + GM CSF plasmid prime and AFP adenoviral vector	Actual enrollment: 2 patients with HCC	Status: terminated (Poor accrual and limited target patient population for future accrual, did not complete the Phase 1 portion of the trial.)	NCT00669136
Oncolytic virus (OVs) based immunotherapy					
Byeong et al. (2008)	JX-594	• A phase 1 clinical trial, assessment of intratumoral injection of JX-594 into primary or metastatic liver tumours • P:safety, MTD	14 patients with primary or metastatic liver tumors	Status: terminated Results: well-tolerated; MTD was determined as 1 × 10^9^ pfu; 10 patients were radiographically evaluable for objective responses; responses in 3 HCC patients: 3 serum tumor markers PR (≥50% decrease); 1 response according to PET	[158] (NCT00629759)
Jeong Heo et al. (2013)	JX-594	• A Prospective, randomized clinical trial with high or low dose JX-594 • P: intrahepatic disease control rate	30 patients with unresectable liver tumors	Status: terminated Results: 11/16 patients showed cytotoxicity against HCC; 31% anorexia in high dose group RR: 4 PR, 10 SD by RECIST	[159] (NCT00554372)
Jennerex Biotherapeutics (2008–2011)	JX-594 (Pexa-Vec)	• A phase 2b randomized trial • JX-594 plus best supportive care versus best supportive care in patients with advanced HCC who have failed Sorafenib treatment	129 patients with advanced HCC who have failed sorafenib	Status: completed Results: none (No results posted on ClinicalTrials.gov)	NCT01387555
SillaJen, Inc. (2015)	Vaccinia virus based immunotherapy (Pexa-Vec) + Sorafenib	• A multi-center, randomized, open-label, Phase 3 trial; • Comparing Vaccinia Virus based Immunotherapy Plus Sorafenib vs Sorafenib alone	600 patients with advanced HCC	Status: recruiting Results: none	NCT02562755

Notes: *HCC* hepatocellular carcinoma, *P* primary endpoint, *S* secondary endpoint, *OS* overall survival, *TTP* time to progression, *TTSP* time to symptomatic progression, *SD* stable disease, *RR* response rate, *JX-594* aoncolyticpox virus carrying human GM-CSF genes, *MTD* maximum-tolerated dose, *RECIST* response evaluation criteria in solid tumors, *PR* partial response

Apart from these classical TAAs, another attractive target is cancer-testis antigens (CTAs), which are considered to be novel targets for HCC immunotherapy due to the restricted expression patterns in a variety of tumors and normal tissues [163]. NY-ESO-1, also known as CTAG1, is one of the most immunogenic CTAs. A number of previous studies reported that NY-ESO-1 is highly expressed in many types of solid tumors, and a number of vaccine strategies targeting NY-ESO-1 are being developed [164–166]. In vitro investigations suggested that NY-ESO-1 expression is associated with poor tumor outcomes, and DCs loaded with NY-ESO-1 peptide can stimulate specific T cell responses against HCC cells [98, 167]. This implies that NY-ESO-1 has potential to be a valuable target molecule for immunotherapy against HCC. Clinically, vaccines targeting NY-ESO-1 expressing solid tumors (including HCC) are ongoing (NCT01522820).

Current studies demonstrate that DNA encoding epitope-optimized murine AFP and lentivector-mediated genetic immunization could induce potent AFP-specific CD8^+^ responses to generate a significant anti-tumor effect in autochthonous HCC model [168]. This may provide additional technology and new perspectives to further maximize the vaccines used in HCC therapy.

Oncolytic viruses (OVs) selectively replicate in tumor cells, damaging them, and subsequently spreading the virus in tumor tissue, while not harming normal cells. This characteristic endows OVs an effective platform for cancer immunotherapy [169]. Pre-clinical and clinical research highlights natural and genetically modified viruses, which are armed with immunomodulatory transgenes, that not only induce potent in situ anti-tumor immunity through mediating immunogenic cell death (ICD) and the inflammatory cascade, but also serve as vectors expressing therapeutic genes to improve cancer treatment [170, 171].

**Fig. 4:**
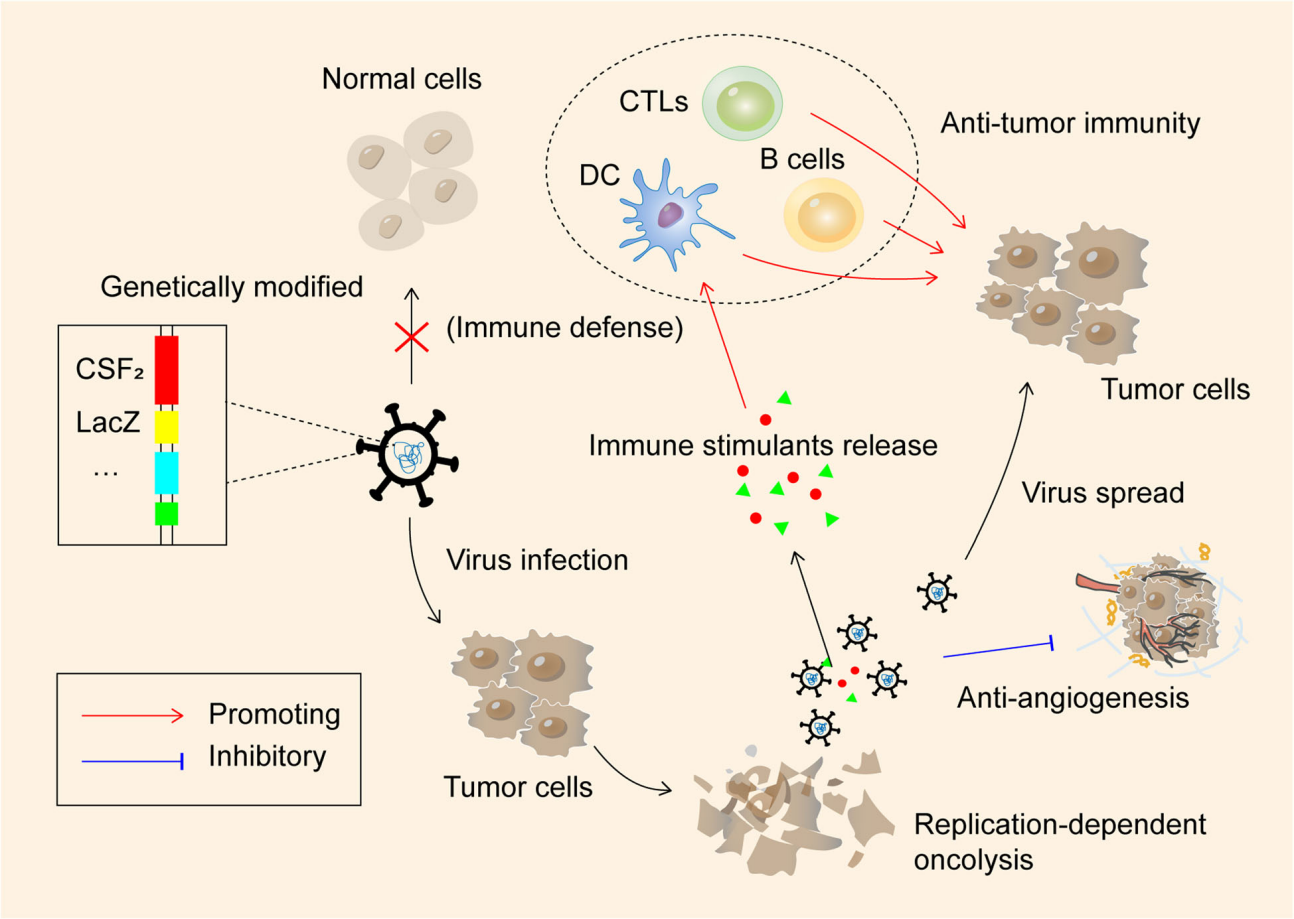
Oncolytic viruses based immunotherapy in HCC**.** Oncolytic viruses (OVs) selectively replicate in and damage tumor cells, subsequently spread in tumor tissue

As for OVs used in HCC immunotherapy, previous in vitro and xenograft mice model studies indicated that a broad variety of therapeutic genes recombinant oncolytic adeno-associated viruses (AAVs) can exert a strong cytopathic effect on HCC cells (Fig. 4**.**). A tumor-selective replicating adenovirus expressing IFN-β, and ZD55-IFN-β, shows an elevated level of IFN-β expression, and 100-fold higher anti-tumor cytotoxicity than replicative adenovirus ONYX-015 [172]. The application of another recombinant AAVs model, AAV vectors containing human telomerase reverse transcriptase (hTERT) and tumor necrosis factor alpha related apoptosis inducing ligand (TRAIL) gene, namely AAV-hTERT-TRAIL, targets telomerase activity in HCC cells, and exhibits specific cytotoxicity and apoptosis to suppress the growth of HCC xenograft tumors [173]. An oncolytic adenovirus coding for granulocyte macrophage colony-stimulating factor (GM-CSF), Ad5-D24-GMCSF, induces tumor-specific and virus-specific immunity both in the syngeneic hamster model and patients. This suggests that oncolytic virus–mediated antitumor immunity may be a promising immunotherapeutic candidate for further clinical testing in HCC treatment [174].

In early clinical studies, oncolytic poxvirus JX-594 (also known as PexaVec), showed robust virus replication-dependent oncolysis, well-tolerated character, anti-vascular effects and anti-tumor immunity in HCC and other solid tumors [158, 175, 176]. (NCT00629759) (Table 5**.**). Another randomized trial in advanced HCC demonstrated oncolytic and immunotherapy mechanisms of action (MOA), tumor responses and dose-related survival (high-dose JX-594 was associated with longer OS) in individuals with HCC [159] (NCT00554372).

In general, OVs is a potent therapeutic agent for cancer treatment, and it’s promising to extend immunotherapeutic options for HCC. Importantly, dosing regimens of OVs must be better defined for its clinical use, and in this regard, further results from clinical trials are awaited.

## Conclusions

HCC is characterized by immune tolerance and comprises numerous infiltrated immune cells, a great number of suppressive molecules, complex pro-inflammatory/immunoregulatory signaling and intricate interactions between different components. The picture of immune microenvironment in HCC plays a key role in HCC progression and recurrence. Apparently, interactions of HCC tumor cells and various immune components in TME are really complicated and multifaceted, finally determining the plasticity and heterogeneity of its both innate and adaptive immune responses. Transcriptional and epigenetical alterations [177], metabolic reprogramming [178] and lack of co-stimulatory signals partially contribute to exhausted phenotype of TILs. Moreover, importantly, the benefit of current predictive biomarkers (e.g. PD-L1 expression level and tumor mutation burden (TMB)) in HCC patients receiving ICBs are still limited. Despite some impressive basic and translational discoveries, more details about underlying cellular or molecular mechanisms of immune evasion in HCC need to be further clarified. It’s clear that a better understanding of HCC immune landscape and will provide new breakthroughs in its clinical treatment.

Recently, immunotherapy brings great promises and new opportunities for HCC therapeutics. Its success has been evidenced by extensive studies. However, a subset of patients with HCC has little positive clinical responses to this treatment. In addition to the current combination regimens of ICBs with TKIs, or individualized cell therapeutic approaches, more effective ways to reinvigorate anti-tumor responses are urgently warranted. In this regard, combination of PD-1/PD-L1 monoclonal antibody and targeting co-stimulatory receptors (such as 4-1BB, OX40, CD27) with agonistic antibodies seems to be a potential therapeutic option for HCC, which may enhance and reverse functions of exhausted CD8^+^ TILs [179]. Additionally, strategies to target altered metabolic characteristics (e.g. the Warburg effect, abnormal glutamine metabolism, and urea cycle deficiency (UCD)) or interfere with the “key point molecules” (e.g. Arginase and indoleamine 2,3-dioxygenase (IDO)) that both influence metabolic reprogramming and T cell exhaustion may be a promising exploration clinically [180]. Moreover, some emerging pre-clinical investigations indicate the developing novel therapeutic approaches like epigenetic therapy using histone deacetylase inhibitors (HDACi) combined with CAR-T treatment are helpful for identification of more precise biomarkers and opening new avenues of HCC immunotherapy.

In general, immunotherapy is becoming one of the most promising approaches for HCC treatment, and it is likely to be more powerful in the foreseeable future.

## Data Availability

Not applicable.

## Abbreviations

ACT: Adoptive cell transfer
AFP: A-fetoprotein
APCs: Antigen presenting cells
CAF: Cancer associated fibroblast
CAR-T: Chimeric antigen receptor-modified T cells
CCL2: Chemokine (C-C motif) ligand 2
CIK: Cytokine-induced killer
CSCs: Cancer stem cells
CTAs: Cancer-testis antigens
CTLA-4: Cytotoxic T lymphocyte protein 4
CTLs: Cytotoxic T lymphocytes
CXCL17: Chemokine (C-X-C motif) ligand 17
CXCR4: Chemokine (C-X-C motif) receptor 4
DCs: Dendritic cells
DEXs: Dendritic cell-derived exosomes
GPC-3: Glypican-3
HCC: Hepatocellular carcinoma
HGF: Hepatocyte growth factor
HSCs: Hepatic stellate cells
hTERT: human telomerase-reverse transcriptase
ICB: Immune-checkpoint blockade
IDO: Indoleamine-2,3-dioxygenase
IFN-γ: Interferon-γ
IL-: Interleukin-
KC: Kupffer cell
LAG-3: Lymphocyte activation gene 3
MAGE-1: Melanoma-associated antigen 1
MCP-1: Monocyte chemotactic protein 1
MDSCs: Myeloid- derived suppressor cells
MTD: Maximum-tolerated dose
NK cell: Natural killer cell
OS: Overall survival
OVs: Oncolytic viruses
P: Primary endpoint
PD-1: Programmed cell death protein-1
PDGF: Platelet-derived growth factor
PD-L1: Programmed cell death protein ligand-1
PR: Partial response
RECIST: Response evaluation criteria in solid tumors
RR: Response rate
S: Secondary endpoint
SD: Stable disease
SDF-1α: Stromal cell derived factor 1α
TAA: Tumor-associated antigens
TAMs: Tumor-associated macrophages
TANs: Tumor associated neutrophils
TEXs: Tumor cell–derived exosomes
TGF-β: Transforming growth factor-β
Tim-3: Mucin domain-containing molecule-3
TME: Tumor microenvironment
Tregs: Regulatory T cells
TTP: Time to progression
TTSP: Time to symptomatic progression
VEGF: Vascular endothelial growth factor
